# Clinical and Immunological Study of 30 Cases With Both IgG and IgA Anti-Keratinocyte Cell Surface Autoantibodies Toward the Definition of Intercellular IgG/IgA Dermatosis

**DOI:** 10.3389/fimmu.2018.00994

**Published:** 2018-05-07

**Authors:** Takashi Hashimoto, Kwesi Teye, Koji Hashimoto, Katarzyna Wozniak, Daisuke Ueo, Sakuhei Fujiwara, Kazuhiro Inafuku, Yorihisa Kotobuki, Ines Lakos Jukic, Branka Marinović, Anna Bruckner, Daisuke Tsuruta, Tamihiro Kawakami, Norito Ishii

**Affiliations:** ^1^Department of Dermatology, Osaka City University Graduate School of Medicine, Osaka, Japan; ^2^Kurume University School of Medicine, Kurume, Japan; ^3^Kurume University Institute of Cutaneous Cell Biology, Kurume, Japan; ^4^Department of Life Sciences, Graduate School of Arts and Sciences, University of Tokyo, Tokyo, Japan; ^5^Department of Dermatology and Immunodermatology, Medical University of Warsaw, Warsaw, Poland; ^6^Ueo Dermatology Clinic, Saiki, Japan; ^7^Department of Dermatology, Faculty of Medicine, Oita University, Yufu, Japan; ^8^Department of Dermatology, Kimitsu Chuo Hospital, Kimitsu, Japan; ^9^Department of Dermatology, Osaka University Graduate School of Medicine, Suita, Japan; ^10^University Hospital Centre Zagreb, Department of Dermatology and Venereology, School of Medicine, University of Zagreb, Zagreb, Croatia; ^11^Pediatric Dermatology, University of Colorado School of Medicine, Children’s Hospital Colorado, Denver, CO, United States; ^12^Department of Dermatology, St. Marriana Medical University, Kawasaki, Japan; ^13^Department of Dermatology, Kurume University School of Medicine, Fukuoka, Japan

**Keywords:** autoimmune bullous diseases, desmocollin, desmoglein, ELISA, intercellular IgG/IgA dermatosis

## Abstract

Several sporadic cases, in which direct and indirect immunofluorescence studies simultaneously detected IgG and IgA autoantibodies to keratinocyte cell surfaces, have been reported mainly under the name of IgG/IgA pemphigus. However, there have been no systematic studies for this condition. In this study, we collected 30 cases of this condition from our cohort of more than 5,000 autoimmune bullous disease cases, which were consulted for our diagnostic methods from other institutes, and summarized their clinical and immunological findings. Clinically, there was no male–female prevalence, mean age of disease onset was 55.6 years, and mean duration before this condition was suspected was 18 months. The patients showed clinically bullous and pustular skin lesions preferentially on the trunk and extremities, and histopathologically intraepidermal pustules and blisters with infiltration of neutrophils and eosinophils. Immunologically, ELISAs frequently detected IgG and IgA autoantibodies to both desmogleins and desmocollins. From the characteristic clinical, histopathological, and immunological features, which are considerably different from those in classical IgG types of pemphigus, we propose this disease as a new disease entity with preferential name of intercellular IgG/IgA dermatosis (IGAD). This was the largest study of IGAD to date.

## Introduction

Autoimmune bullous disease (AIBD) is divided into pemphigus group with autoantibodies to keratinocyte cell surfaces (CSs) and pemphigoid group with autoantibodies to epidermal basement membrane zone (BMZ) (1, 2). Two representative classical IgG types of pemphigus are pemphigus vulgaris (PV) and pemphigus foliaceus (PF), which react with desmoglein 3 (Dsg3) and Dsg1, respectively, although there are many other forms of pemphigus (3, 4). Among them, cases with anti-CS antibodies exclusively of IgA class had been called as IgA pemphigus (5–8), for which we proposed intercellular IgA dermatosis (IAD) as a preferable name (5, 7, 8).

IgG/IgA pemphigus is the name given to an atypical form of pemphigus characterized by *in vivo* bound and/or circulating anti-keratinocyte CS antibodies of both IgG and IgA classes (1). The results in approximately 20 reports indicated that IgG/IgA pemphigus is an atypical form of pemphigus with heterogeneous clinical and histopathological features (9–30). However, because this condition is extremely rare, there is no systematic study and disease entity of this condition has not been established. At Kurume University, we have examined more than 5,000 cases of various AIBDs, which were consulted at other institutes for our diagnostic studies (1, 31). Therefore, in this study, we attempted to determine the characteristic clinical, histopathological, and immunological features of all patients with both IgG and IgA anti-keratinocyte CS antibodies as the first step to establish this disease entity.

In this retrospective study, we selected 30 cases with IgG and IgA anti-CS antibodies from our AIBD cohort, and characterized them clinically, histopathologically, and immunologically. Considerably distinct features found in these cases indicated this condition as a new disease entity, and we propose the term “intercellular IgG/IgA dermatosis (IGAD)” to this disorder, following the designation of IAD.

## Materials and Methods

This study was performed following Declaration of Helsinki and guidelines of local ethics committees of Kurume University School of Medicine. Informed consents were provided by all patients and normal individuals.

### Cases and Sera

In this study, we used our AIBD cohort of 5,402 cases. Information and sera for these cases were sent to us from other institutes in either Japan or other countries between 1996 and 2015. Information of clinical and histopathological findings and direct immunofluorescence (IF) was obtained from consultation letters.

### Various Immunological Methods

#### IF Studies

Direct IF for IgG, IgA, IgM, and C3 using skin biopsies was performed mainly at other institutes. Indirect IF studies of normal human skin and monkey esophagus for both IgG and IgA antibodies were performed by standard method. In cases with autoimmune reactivity with epidermal BMZ, indirect IF of 1 M NaCl-split normal human skin for IgG and IgA antibodies were also performed (32).

#### Immunoblotting Studies

Immunoblotting of normal human epidermal extract was performed as described previously (33, 34). In cases with reactivity with BMZ, we also performed IB analyses using BP180 NC16a domain recombinant protein (RP) (35), BP180 C-terminal domain RP (36), concentrated culture supernatant of HaCaT cells (37), normal human dermal extract (38), and purified human laminin-332 (39) for both IgG and/or IgA antibodies.

#### ELISA Studies

Commercially available IgG ELISAs of Dsg1 and Dsg3 (cutoff: <index 12) (MESACUP, MBL, Nagoya, Japan) (40) were conducted according to the manufacturer’s instruction. Using the same ELISA kits, IgA antibodies to Dsg1 and Dsg3 (cutoff: <OD 0.15) were also examined (41). In addition, ELISAs of mammalian RPs of human desmocollin 1 (Dsc1)-Dsc3 were performed for both IgG antibodies (42) and IgA antibodies (43). Cutoff OD values were 0.2 for Dsc1, 0.07 for Dsc2, and 0.12 for Dsc3 for IgG antibodies, and 0.123 for Dsc1, 0.048 for Dsc2, and 0.074 for Dsc3 for IgA antibodies. Furthermore, in cases with reactivity with BMZ, we performed IgG ELISAs of BP180 NC16a domain RP (cutoff: <index 15) (44), BP230 RPs (cutoff values: <index 9) (45), and type VII collagen RP (MBL) (46). OD at 490 nm was measured by ELISA reader.

### COS-7 Cell cDNA Transfection Method

COS-7 cell cDNA transfection method using cDNAs of human Dsc1–Dsc3 was performed as described previously (47).

### Statistical Analyses

We statistically analyzed correlations between the results in Dsg and Dsc ELISAs and clinical parameters. Differences among qualitative results were compared using the chi-square test. Differences among quantitative parameters between groups were assessed using the Mann–Whitney *U* test. *p* Values less than 0.05 were considered significant.

## Results

### Diagnoses

In this study, we suspected the diagnosis of IGAD for 30 cases, which showed simultaneous IgG and IgA immunoreactivity with keratinocyte CS antigens in direct IF and various serological studies (whole clinical and immunological data are shown in Tables S1 and S2 in Supplementary Material, respectively). In addition, four cases showed IgG and/or IgA anti-BMZ immunoreactivity by IF and/or molecular studies.

Clinical diagnosis in the consultation letters for the 30 cases included IGAD (12 cases), IAD (9 cases), and other AIBDs (7 cases), while no clinical diagnosis was given in 2 cases (Table S3A in Supplementary Material).

Regarding the diagnoses after the characterization, 26 IGAD cases had sole IGAD, while bullous pemphigoid, linear IgA bullous dermatosis, and linear IgA/IgG bullous dermatosis were concomitant in 1, 1, and 2 cases, respectively (Table S3B in Supplementary Material). In addition, one case each was suggested to be named as IgG/IgA PF and IgG/IgA PV.

Because of the large cohort size and long surveillance time, most of the clinical, histopathological, and immunological results were not obtained from all cases. Therefore, we summarize only available results for each parameter in the following sections.

### Detailed Reports for Six New IGAD Cases

**Figure 1:**
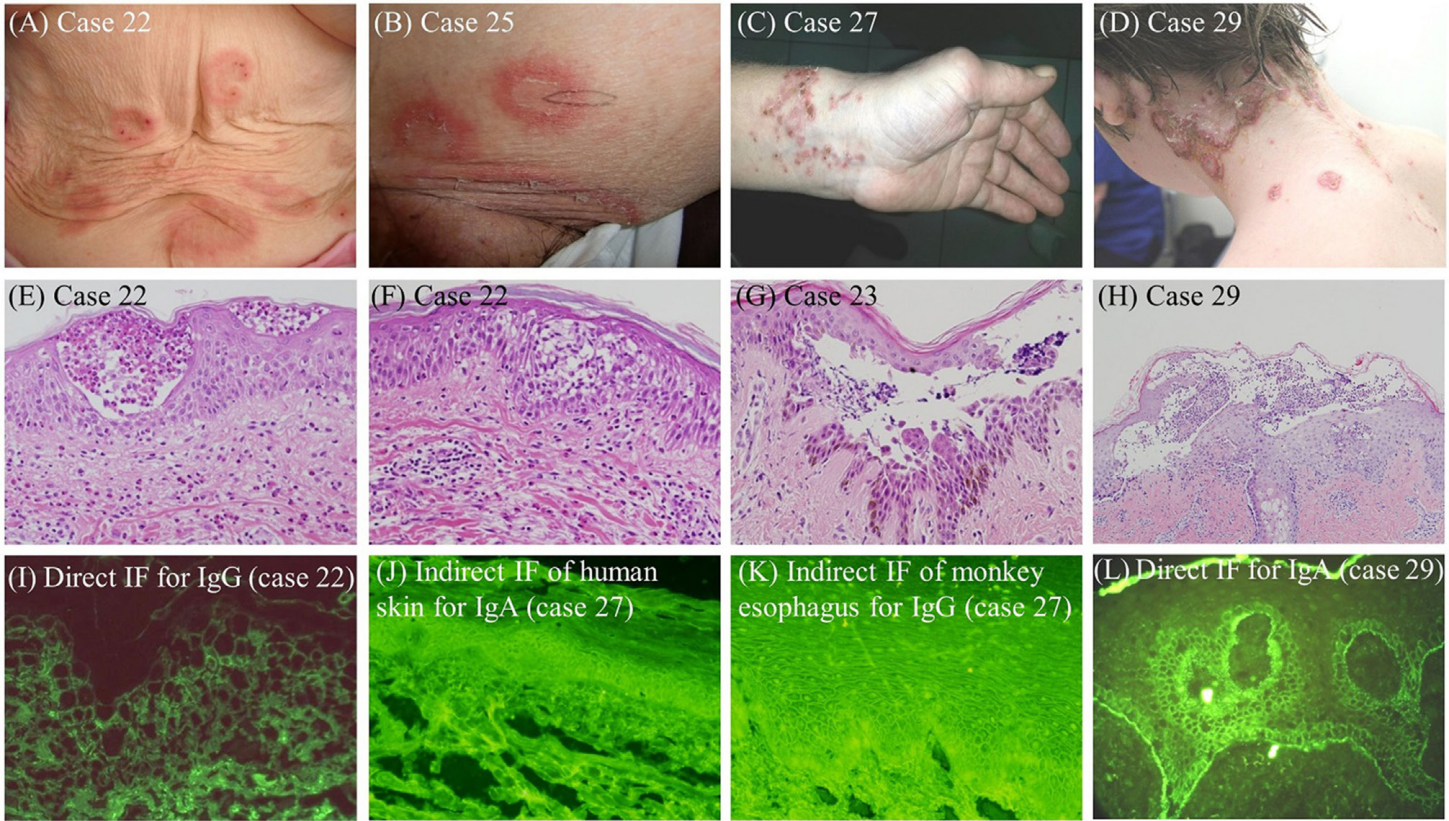
Clinical **(A–D)**, histopathological **(E–H)**, and IF **(I–L)** features in representative IGAD cases examined in this study. **(A)** Vesicles were seen on the peripheries of annular erythemas on the abdomen (case 22). **(B)** Annular erythemas with superficial pustules and desquamation were seen (case 25). **(C)** Erythematous skin lesions with pustules and crusts on the left wrist were seen (case 27). **(D)** Pustular skin lesions on the peripheries of annular erythemas were seen (case 29). **(E,F)** Eosinophilic pustules in the upper epidermis **(E)** and eosinophilic spongiosis in the middle epidermis **(F)** were seen (case 22). **(G)** Acantholytic blisters and pustules in the middle epidermis were seen (case 23). **(H)** Subcorneal pustules with predominant neutrophils and a few eosinophils were seen. **(I)** The result of direct IF for IgG (case 22). **(J)** The result of indirect IF of normal human skin for IgA antibodies (case 27). **(K)** The results of indirect IF of monkey esophagus for IgG antibodies (case 27). **(L)** The result of direct IF for C3 (case 29).

Clinical and histopathological findings and disease courses for six patients are described in Supplementary data (cases 17, 22, 23, 25, 27, and 29 in Tables S1 and S2 in Supplementary Material), as well as corresponding figures (Figures S1–S6 in Supplementary Material). Clinical, histopathological, and IF features in representative IGAD cases are also depicted in Figure 1.

### Clinical Findings in 30 IGAD Cases

#### Backgrounds of the Cases

The 30 IGAD cases included 18 Japanese, 6 European, 3 US, 2 Indian, and 1 Australian. There were 15 males and 13 females, with 2 cases without information of gender. Mean ages of 30 cases were 55.6 years (53.4 years for males and 57.8 years for females). The durations between the disease onset and the consultation to us for 21 cases were from 2 weeks to 10 years (mean 18 months).

Before the cases visited the practitioners, who consulted us, one each case was treated with systemic steroid, antihistamine, combination of systemic steroid and antihistamine, and minocycline. Administration of vancomycin was not reported.

Underlying diseases included six malignant tumors, i.e., two cases of lung cancer and one case each of uterus carcinoma, breast carcinoma, gallbladder carcinoma, and malignant lymphoma. In addition, one case each had diabetes mellitus, IgA nephropathy, myasthenia gravis, Sweet’s syndrome, Sjogren’s syndrome, and PV.

#### Clinical Manifestations

Involved sites available from consultation letters for the 30 IGAD cases were the whole body (2 cases), the trunk (19 cases), the extremities (17 cases), the intertriginous areas (4 cases), and the head/face (6 cases) (Figure 1; Table S1 in Supplementary Material). Mucosal lesions were found in oral mucosae (11 cases) and conjunctiva (2 cases), with 1 case each with genital, nasal, and esophageal mucosae.

Regarding clinical manifestations, 19 cases showed blister formation with vesicles (5 cases), tense bullae (2 cases) and flaccid bullae (3 cases), 10 cases showed pustule formation, 10 cases showed erosions, and 17 cases showed erythemas with annular erythemas (9 cases). Other clinical manifestations were crust formation (3 cases), pigmentation (2 cases), and vegetating lesions (1 case). Itch was complained by 6 patients.

#### Histopathological Features

Among 23 IGAD cases with histopathological information for skin biopsies, 10 cases showed intraepidermal blister formation at the upper epidermis or middle epidermis, while 12 cases showed intraepidermal pustules at the upper epidermis, middle or lower epidermis (Figure 1; Table S1 in Supplementary Material). Spongiosis and acantholysis were observed in three and four cases, respectively. Intraepidermal infiltrations of neutrophils, eosinophils, and lymphocytes were found in 11, 8, and 4 cases, respectively. Presence and absence of acantholysis were described in one and three cases, respectively. Two cases showed subepidermal blisters.

#### Treatments, Responsiveness, and Complications

Sole therapy of oral steroids, dapsone (DDS), minocycline, and combinations of these drugs and other treatments were performed as described in 13 cases (Tables S1 and S4 in Supplementary Material). The responsiveness to these therapies was variable (Table S4 in Supplementary Material).

### Immunological Findings

#### Direct IF

The results of direct IF were described in the consulting letters for 22 patients. IgG, IgA, and C3 depositions to keratinocyte CSs were positive in 17, 17, and 5 cases, respectively (Table 1A). IgG deposition was seen at upper epidermis in one case, lower epidermis in two cases, and entire epidermis in two cases. IgA deposition was seen at upper epidermis in one cases, lower epidermis in four cases, and entire epidermis in four cases. IgG, IgA, and C3 depositions to epidermal BMZ were observed in one, one, and two cases, respectively.

**Table 1:** Results of various immunofluorescence (IF) studies.

		Positive	Total	Positive rates (%)
**(A) Direct IF for skin biopsy**				
Cell surface (CS)	IgG	17	22	77.27
Upper epidermis		1		5.88
Lower epidermis		2		11.76
Entire epidermis		2		11.76

CS	IgA	17	22	77.27
Upper epidermis		1		5.88
Lower epidermis		4		23.53
Entire epidermis		4		23.53

CS	C3	5	6	83.33

BMZ	IgG	1	21	4.76
	IgA	1	21	4.76
	C3	2	5	66.67

**(B) Indirect IF using normal human skin as a substrate**				
IgG	CS	17	29	58.6
IgA	CS	20	29	69.0

**(C) Indirect IF using monkey esophagus as a substrate**				
IgG	CS	14	18	77.8
IgA	CS	14	18	77.8

**(D) Indirect IF using 1 M NaCl-split normal human skin as a substrate**				
IgG	Epidermal side	2	16	12.5
	Dermal side	0	16	0.0

IgA	Epidermal side	3	16	18.8
	Dermal side	0	16	0.0

*CS, keratinocyte cell surface; BMZ, basement membrane zones*.

#### Indirect IF

In indirect IF of normal human skin performed for 29 IGAD cases, IgG and IgA anti-keratinocyte CS antibodies were positive in 17 and 20 cases, respectively (Figure S3 in Supplementary Material) (Table 1B–D). In addition, two and one cases showed IgG and IgA anti-BMZ antibodies, respectively. Indirect IF of monkey esophagus performed for 18 cases detected anti-epithelial CS antibodies of both IgG and IgA classes in 14 cases. In addition, two cases each showed IgG and IgA anti-BMZ antibodies. In indirect IF of 1 M NaCl-split human skin performed for 16 cases, IgG and IgA reactivity with epidermal side was found in two and three cases, respectively.

#### IB Studies of Normal Human Epidermal Extract and Other Substrates for BMZ Autoantigens

In immunoblotting of normal human epidermal extract performed for all 30 IGAD cases, few cases showed positive reactivity with various epidermal autoantigens for both IgG and IgA classes (Table S5 in Supplementary Material). For IgG antibodies, three cases each reacted with Dsg1 and Dsg3 and one case each reacted with both a and b forms of Dsc. One case each reacted with desmoplakin I, BP230, and envoplakin. For IgA antibodies, one case reacted with Dsg3 and two cases each reacted with both a and b forms of Dsc.

The four cases with positive reactivity with BMZ in IF studies were further examined by immunoblotting for the reactivity with various BMZ autoantigens. Two cases showed positive IgG, but not IgA, reactivity with BP180 NC16a domain RP, while none reacted with BP180 C-terminal domain RP for either IgG or IgA antibodies. One case was positive for IgG, but not IgA, for the 120-kDa LAD-1 in concentrated culture supernatant of HaCaT cells. No positive reactivity was found in immunoblotting of both purified human laminin-332 and normal human dermal extracts for either IgG or IgA antibodies.

### ELISAs

In commercially available IgG ELISAs for Dsg1 and Dsg3 for all 30 cases, 19 (63.6%) and 14 (46.7%) cases showed IgG reactivity with Dsg1 and Dsg3, respectively, and 19 (63.3%) and 13 (43.3%) cases showed IgA reactivity with Dsg1 and Dsg3, respectively (Table 2A). In ELISAs of mammalian RPs of Dsc1–Dsc3, 6 (20.0%), 7 (23.3%), and 11 (36.7%) cases showed IgG reactivity with Dsc1, Dsc2, and Dsc3, respectively, while 5 (16.7%), 8 (26.7%), and 7 (23.3%) cases showed IgA reactivity with Dsc1, Dsc2, and Dsc3, respectively (Table 2B).

**Table 2:** Results of various ELISAs and COS7 cell cDNA transfection.

	Positive	Total	Positive rates (%)
**(A) ELISAs for Dsg1 and Dsg3 for IgG and IgA antibodies**			
ELISA Dsg1 (IgG)	19	30	63.3
ELISA Dsg3 (IgG)	14	30	46.3
ELISA Dsg1 (IgA)	19	30	63.3
ELISA Dsg3 (IgA)	13	30	43.3

**(B) ELISAs for desmocollin 1 (Dsc1)-Dsc3 for IgG and IgA antibodies**			
ELISA Dsc1 (IgG)	6	30	20.0
ELISA Dsc2 (IgG)	7	30	23.3
ELISA Dsc3 (IgG)	11	30	36.7
ELISA Dsc1 (IgA)	5	30	16.7
ELISA Dsc2 (IgA)	8	30	26.7
ELISA Dsc3 (IgA)	7	30	23.3

**(C) COS-7 cell cDNA transfection methods for Dsc1-Dsc3 for IgG and IgA antibodies**			
COS-7 Dsc1 (IgG)	0	23	0.0
COS-7 Dsc2 (IgG)	0	23	0.0
COS-7 Dsc3 (IgG)	1	23	4.3
COS-7 Dsc1 (IgA)	1	24	4.2
COS-7 Dsc2 (IgA)	0	24	0.0
COS-7 Dsc3 (IgA)	2	24	8.3

Interestingly, there were very high association of detection of IgG and IgA antibodies to the same Dsgs, i.e., 18 (94.7%) of 19 cases with IgG antibodies to Dsg1 had IgA antibodies to Dsg1, 18 (94.7%) of 19 cases with IgA anti-Dsg1 antibodies had IgG anti-Dsg1 antibodies, 11 (78.6%) of 14 cases with IgG anti-Dsg3 antibodies had IgA anti-Dsg3 antibodies, and 11 (84.6%) of 13 cases with IgA anti-Dsg3 antibodies had IgA anti-Dsg3 antibodies. By contrast, rates of coexistence of IgG and IgA antibodies to Dsc1–Dsc3 were not very high, i.e., only 50.0% (3/6), 57.1% (4/7), and 54.5% (6/11) of cases with IgG antibodies to Dsc1, Dsc2, and Dsc3 had IgA antibodies to the same Dscs (Table S2 in Supplementary Material).

One of the four cases with positive reactivity with BMZ in IF studies was positive in IgG ELISA of BP180 NC16a RP (data not shown).

#### COS-7 Cell cDNA Transfection Methods

In COS-7 cell cDNA transfection method performed for 23 and 24 cases for IgG and IgA antibodies, respectively, one case showed IgG reactivity with Dsc3, and one and two cases showed IgA reactivity with Dsc1 and Dsc3, respectively (Table 2C).

#### Statistical Analyses Between the Dsg and Dsc ELISAs and Clinical Parameters

The case with positive reactivity with Dsg3 for both IgG (*p* = 0.0103) and IgA antibodies had oral mucosal lesions significantly more frequently. Female cases had both IgA anti-Dsg1 antibodies (*p* = 0.0351) and IgG anti-Dsc1 antibodies (*p* = 0.0409) more frequently (chi-square for independence test). No statistically significant correlations were found for any other clinical parameters, including ages, disease durations, sites and manifestations of skin lesions, and responses to various treatments, as well as histopathological features.

## Discussion

In this study, although the diagnosis of IGAD was suspected by atypical clinico-histopathological features, we made possible diagnosis of IGAD for patients showing simultaneously IgG and IgA reactivity in various immunological examinations, including IF, immunoblotting, ELISA, and cDNA transfection studies. Particularly, ELISAs were sensitive and detected IgG and IgA antibodies to Dsgs and Dscs in considerable numbers of patients. Finally, using the tentative diagnostic criteria, we diagnosed 30 patients as IGAD.

Clinically, skin lesions developed mainly on the trunk and extremities, and 11 cases showed oral mucosal lesions. Most patients showed blisters, erosions, and/or pustules with erythematous lesions. In most cases, histopathology showed intraepidermal blister and/or pustules at various layers in the epidermis. The major infiltrating cells were neutrophils, followed by eosinophils and lymphocytes. Acantholysis was not clearly seen in some of the patients. Spongiosis were seen occasionally. These clinico-histopathological findings were generally compatible with those in previously reported cases (9–30). In 11 cases, various therapies, mainly oral steroids and DDS, were performed with different efficacy.

Immunologically, direct IF and indirect IF of normal human skin and monkey esophagus were the most sensitive methods with positive rates from 60 to 80%. Regarding antigen detection methods, IgG and IgA ELISAs for Dsgs and Dscs were very sensitive. The IgG and IgA ELISAs detected antibodies to Dsg1 and Dsg3 in 63 and 45% of the patients, respectively, and antibodies to Dsc1–Dsc3 in 20–40% of the patients. Because anti-Dsc antibodies are rarely detected in classical IgG type pemphigus diseases (42), the frequent detection of anti-Dsc antibodies for both IgG and IgA antibodies was considered the significant feature in IGAD. The presence of autoantibodies to other than Dsgs may account for the absence of acantholysis in some patients.

By contrast, sensitivity of immunoblotting of normal epidermal extract and cDNA transfection was very low.

Statistical analyses revealed that anti-Dsg3 antibodies of both IgG and IgA classes were more frequently detected in the cases with oral mucosal lesions. Because Dsg3 is autoantigens found in mucosal-type PV, the results further suggested the significant involvement of IgG anti-Dsg3 antibodies in oral involvement in IGAD. This result may also suggest the pathogenic role of IgA anti-Dsg3 antibodies in development of oral lesions. However, because of the high rates of simultaneous detection of IgG and IgA anti-Dsg3 antibodies in the same sera, the oral lesions might be produced by IgG, but not IgA, anti-Dsg3 antibodies.

Thus, IGAD patients tended to show clinico-histopathological features of both blisters and pustules. IgG-type pemphigus usually shows blisters, while IAD shows pustules. Therefore, IGAD seems to show mixed features of IgG-type pemphigus and IAD. Immunologically, IgG and IgA antibodies tended to react with both Dsgs and Dscs, which also indicates mixed immunological features of IgG-type pemphigus and IAD. These clinical, histopathological, and immunological features are different from either IgG-type pemphigus or IAD. Therefore, we concluded that IGAD is a distinct clinical entity with unique clinical and immunological features.

In this study, we performed no disease model experiments for the pathogenic role of IgG and IgA antibodies in IGAD. However, several IGAD cases showed clear acantholytic histology, suggesting that IgG antibodies to Dsg1 and/or Dsg3 induced the cell detachment similar to IgG-type pemphigus. In addition, because most IGAD cases showed extensive pustular lesions, IgA antibodies to either Dsgs or Dscs may produce the pustular lesions similar to IAD.

We should also consider the molecular mechanisms of simultaneous production of autoantibodies of IgG and IgA classes to various keratinocyte CS antigens, particularly class switch recombination (CSR) for antibody class switching (48, 49). CSR occurs through a genomic rearrangement within constant region locus of immunoglobulin heavy chain, where gene segments of all immunoglobulin classes are tandemly located downstream of the VDJ variable region locus (Figure S7 in Supplementary Material). The upstream classes are looped out through the CSR and the downstream class is docked into the VDJ region (Figure S7 in Supplementary Material). Thus, the class switching is irreversible and a class must be switched from left to right. Although a class has been considered to be switched from IgM/IgD-producing B-cells to IgG-, IgA-, and IgE-producing B-cells (50), a recent study of comprehensive antibody repertoire sequencing followed by lineage tracing revealed more variable class switch pathways, including pathway from IgG1 to IgA1 (Figure S7 in Supplementary Material) (51).

The extremely high rates of simultaneous detection of IgG and IgA anti-Dsg3 antibodies in the same sera found in this study may indicate that IgG-producing B-cells converted to IgA-producing B-cells. Although we could not determine the ancestral class, predominance of IgG1 class in human serum may suggest that the autoantibody switch from IgG1 to IgA1. By contrast, concurrence of IgG and IgA antibodies to the same Dscs was not very high, suggesting that different immunological IgG and IgA autoantibodies may develop by different molecular mechanisms between Dsgs and Dscs. In future studies, these CSR-related molecular mechanisms in IGAD should be examined more extensively using subclass-specific secondary antibodies or methods to detect the epitopes in more detail.

In conclusion, the present study was the first systematic study for IGAD, and suggested that IGAD is a distinct disease entity with characteristic clinical, histopathological, and immunological features. Combination methods of direct IF, indirect IF of normal human skin/monkey esophagus and ELISAs of Dsgs and Dscs are useful to make the diagnosis of IGAD.

## Author Contributions

TH, DT, and NI designed the study. KW, DU, SF, KI, YK, IJ, BM, AB, and TK gave clinical and histopathological information of the patients. KT and NI performed the experiments. TH, DT, KT, and NI analyzed the data. TH and KH prepared the figures. TK performed the statistical analyses. TH, KT, KH, DT, and NI wrote the paper.

## Conflict of Interest Statement

The authors declare that the research was conducted in the absence of any commercial or financial relationships that could be construed as a potential conflict of interest. The reviewer MD declared a past co-authorship with one of the authors KW to the handling Editor.
