# Supplementary material for: Clinical and Immunological Study of 30 Cases With Both IgG and IgA Anti-Keratinocyte Cell Surface Autoantibodies Toward the Definition of Intercellular IgG/IgA Dermatosis
Source: Front Immunol. 2018 May 7;9:994. doi: 10.3389/fimmu.2018.00994 (PMC5950707; doi:10.3389/fimmu.2018.00994)
Supplement: Supplementary file 1 [file presentation_1.PDF]

SUPPLEMENTARY DATA

Reports of 6 new cases of intercellular IgG/IgA dermatosis (IGAD)

Case 17 (FIGURE S1)

A 58-year-old female with a past history of breast cancer 5 years ago, who was treated with amlodypine for hypertension, developed reluctant skin lesions, and visited University Hospital of Warsaw School of Medicine, Warsaw, Poland, in October 2010.

Physical examination revealed blisters on the peripheries of annular/herpetiform erythemas on the fingers, hands, forearms and thighs (Figure S1A,B). Mucous membranes were spared.

Histopathology for skin biopsy showed intraepidermal acantholytic blisters with infiltration of neutrophils and lymphocytes in the lower epidermis, and lymophocytic infiltration in the dermis (Figure S1C). Direct immunofluorescence (IF) showed strong deposition of both IgG and IgA to keratinocyte cell surfaces.

In seroimmunological tests, indirect IF of normal human skin detected IgG (1:10), but not IgA, antibodies to keratinocyte cell surfaces. Indirect IF of monkey esophagus showed IgG, but not IgA, anti-epithelial cell surface antibodies.

Immunoblotting (IB) of normal human epidermal extract showed IgG reactivity with the 130 kDa desmoglein 3 (Dsg3), but no reactivity for IgA antibodies.

IgG ELISAs of recombinant proteins (RPs) of human Dsg1 and Dsg3 (MBL, Nagoya, Japan) showed positive results for both Dsg1 (index 61.03, cut-off <14) and Dsg3 (index 145.77, cut-off <7), and IgA ELISAs for Dsg1 and Dsg3 were also positive for both Dsg1 (OD 0.968, cut-off <0.15) and Dsg3 (OD 0.88, cut-off <0.15). IgG and IgA ELISAs of mammalian RPs of human Dsc1-Dsc3 revealed that IgG antibodies were negative all for Dsc1 (OD 0.031, cut-off <0.2), Dsc2 (OD 0.021, cut-off <0.07) and Dsc3 (OD 0.02, cut-off <0.12), while IgA antibodies were negative for Dsc1 (OD 0.046, cut-off <0.123), weakly positive for Dsc2 (OD 0.053, cut-off <0.048) and negative for Dsc3 (OD 0.054, cut-off <0.074).

COS7 cell cDNA transfection method for human Dsc1-Dsc3 for both IgG and IgA antibodies did not show any positive reactivity.

From these results, the diagnosis of IGAD was made. Treatment with oral prednisolone (PSL) 30mg (0.5mg/kg)/day with dapsone 100mg/day lead to prompt

resolution of the skin lesions. No recurrence of both skin lesions and breast cancer was observed during 2 year follow-up period.

FIGURE S1 (Case 17)

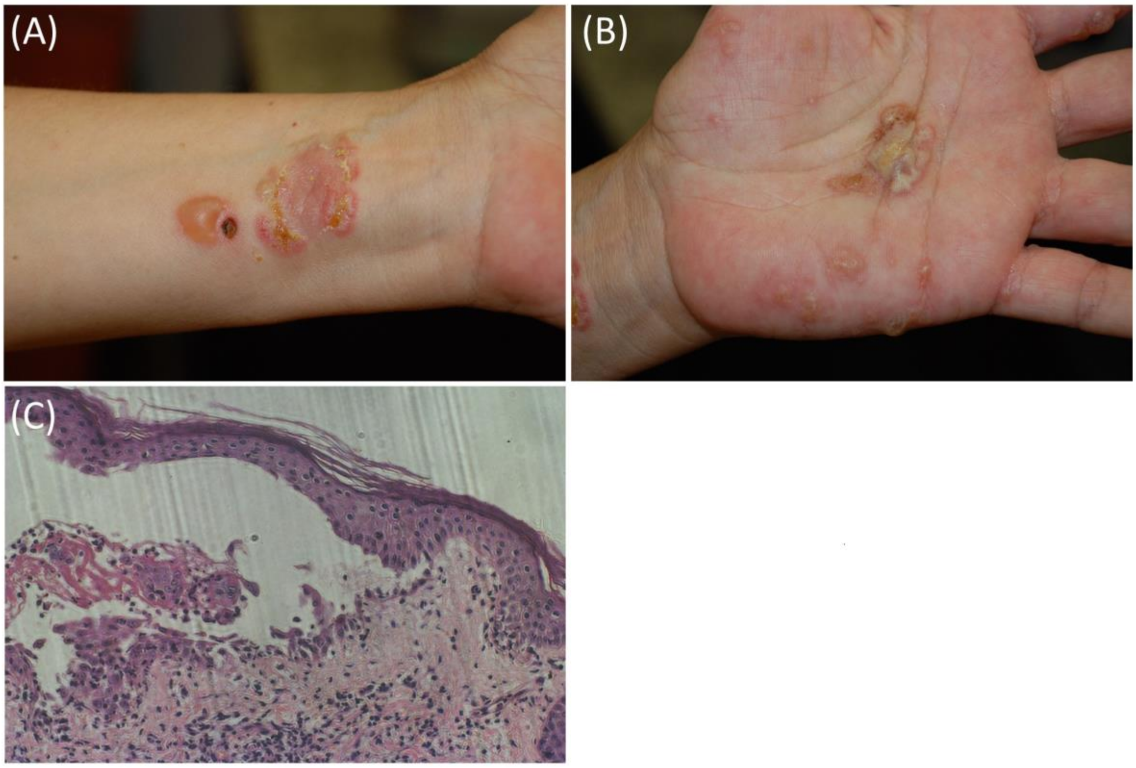

Clinical and histopathological findings in case 17.
(A,B) Clinical features. (C) Histopathological feature.

Case 22 (FIGURE S2)

An 80-year-old Japanese female with a past history of Sjogren’s syndrome, visited Kimitsu Chuo Hospital, Chiba, Japan, in January 2012, complaining of annular erythemas on the trunk and expremities, which had been lasting for 10 days.

Physical examination revealed multiple annular erythemas in various sizes with a few vesicles on the peripheries of the erythemas on the chest, abdomen and extremities (Figure S2A-D). Laboratory examination showed no particular abnormal results, except for mild anemia and slightly elevated CRP. Commercial IgG ELISAs for Dsg1, Dsg3 and BP180 (MBL) showed negative results.

Under suspected diagnosis of annular erythema due to Sjogren’s syndrome, dermatitis herpetiformis Duhring, linear IgA bullous dermatosis and intercellular IgA dermatosis (IAD, also called IgA pemphigus), biopsies were taken from lesional skins on the chest, abdomen and left thigh. Histopathology of biopsies from the abdomen and thigh showed eosinophilic pustules in the upper epidermis (Figure S2E) and eosinophilic spongiosis in the middle epidermis (Figure S2F), as well as eosinophilic infiltration in dermis (Figure S2E,F). There were only few neutrophils, and no acantholytic cells were seen.

Direct IF of biopsy from the chest revealed deposition of IgG (Figure S2G) and C3 (Figure S2H), but not IgA (Figure S2I), to keratinocyte cell surfaces, being stronger in the lower epidermis.

Regarding serological tests, no reactivity for both IgG nor IgA antibodies was detected either in indirect IF of both normal human skin and monkey esophagus or in IB of normal human skin extract. Repeated IgG ELISAs of Dsg1 and IgA ELISAs for Dsg1 and Dsg3 were all negative. IgG ELISAs of Dsc1-Dsc3 were relatively weakly positive for Dsc1 (OD 0.454), negative for Dsc2 (OD 0.016) and strongly positive for Dsc3 (OD 2.254), while IgA ELISAs of Dsc1-Dsc3 were negative for Dsc1 (OD 0.057) and Dsc2 (OD 0.016) but positive for Dsc3 (OD 0.321).

COS7 cell cDNA transfection method of human Dsc1-Dsc3 for both IgG and IgA antibodies did not show any positive reactivity.

From the results of serological tests, the patient was diagnosed as IGAD with major reactivity with Dsc3 for both IgG and IgA antibodies. Oral PSL 20mg/day started, resulting in dramatic improvement of skin lesions within 3 weeks. Then, PSL tapered, and no recurrence was observed upon PSL 5 mg/day during 6 month follow-up period.

FIGURE S2 (Case 22)

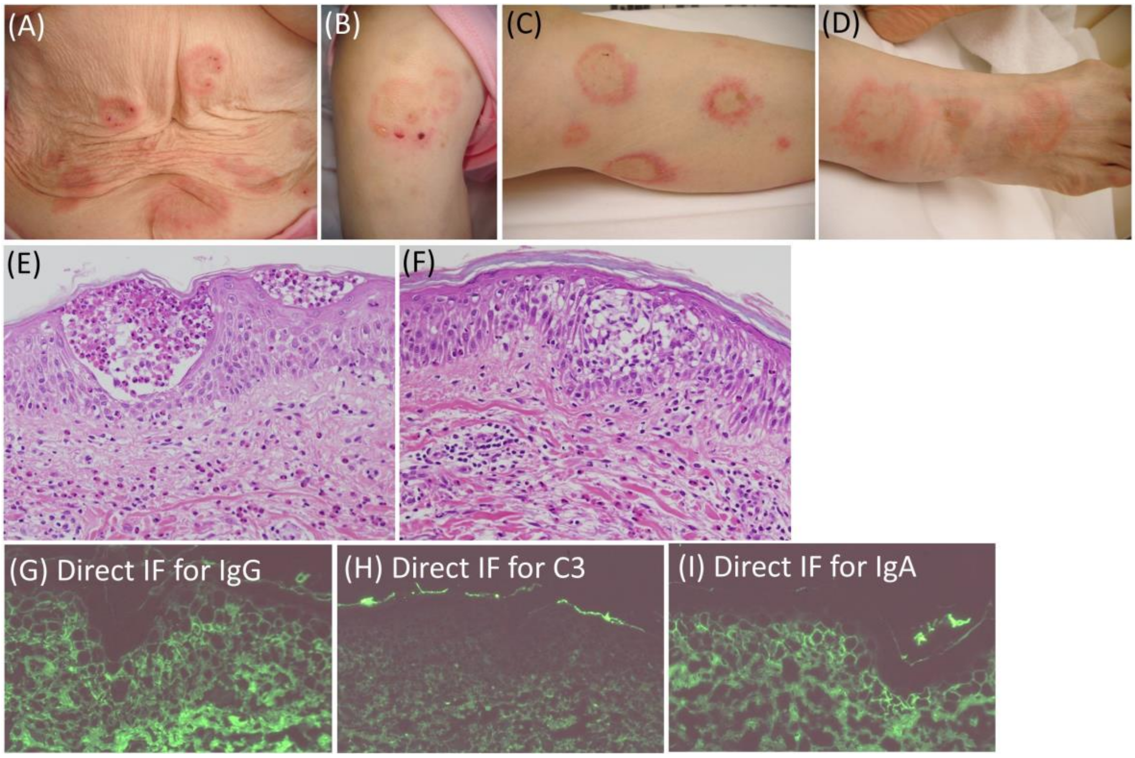

Clinical, histopathological and IF findings in case 22.

(A-D) Clinical features. (E,F) Histopathological features. (G-I) The results of direct IF for IgG (G), C3 (H) and IgA (I).

Case 23 (FIGURE S3)

A 64-year-old Japanese male with no past history had pruritic erythemas sized around 10 mm on the waist and back for about 10 years, which recurred many times under the treatment of topical corticosteroids and anti-histamines. Because the erythematous lesions spread to the trunk and extremities and bullous lesions appeared on the back, the patient visited Osaka University Hospital, Osaka, Japan, in July 2012, and then hospitalized.

Physical examination revealed pruritic erythematous skin lesions with tense vesicles on the trunk and extremities (Figure S3A-D). Post-inflammatory pigmentations were also observed on the trunk. No oral mucosal lesion was seen. Laboratory investigation did not show abnormal results, except for elevated serum IgE (291.41 unit/ml). The results of all tumor makers tested were within normal ranges. Chest X-ray photography and CT scan for entire body revealed no malignant tumor.

Histopathology for skin biopsy showed either blisters with acantholytic cells or pustules with prominent neutrophils and less numbers of lymphocytes and eosinophils in the entire epidermis (Figure S3E-G). Minimum perivascular inflammatory cell infiltration was seen in the dermis. Direct IF showed deposition of both IgG and IgA to keratinocyte cell surfaces in the entire epidermis.

In seroimmunological studies, indirect IF of normal human skin showed anti-cell surface antibodies for both IgG (1:10) and IgA (1:40) classes, and indirect IF of monkey esophagus also showed both IgG and IgA anti-cell surface antibodies. IB of normal human epidermal extract showed negative results for both IgG and IgA antibodies.

In ELISAs, IgG antibodies were negative for Dsg1 (index 8.99) but positive for Dsg3 (index 8.07), while IgA antibodies were positive for Dsg1 (OD 0.193) but negative for Dsg3 (OD 0.011). In ELISAs of mammalian RPs of human Dsc1-Dsc3, IgG antibodies were negative for all Dsc1 (0.097), Dsc2 (0.007) and Dsc3 (0.015), and IgA antibodies were negative for all Dsc1 (0.064), Dsc2 (0.033) and Dsc3 (0.011).

From these findings, the diagnosis of IGAD was made. Oral dapsone (DDS) 50 mg/day controlled well the skin lesions with occasional minimum recurrences during 3 year-follow-up period.

FIGURE S3 (Case 23)

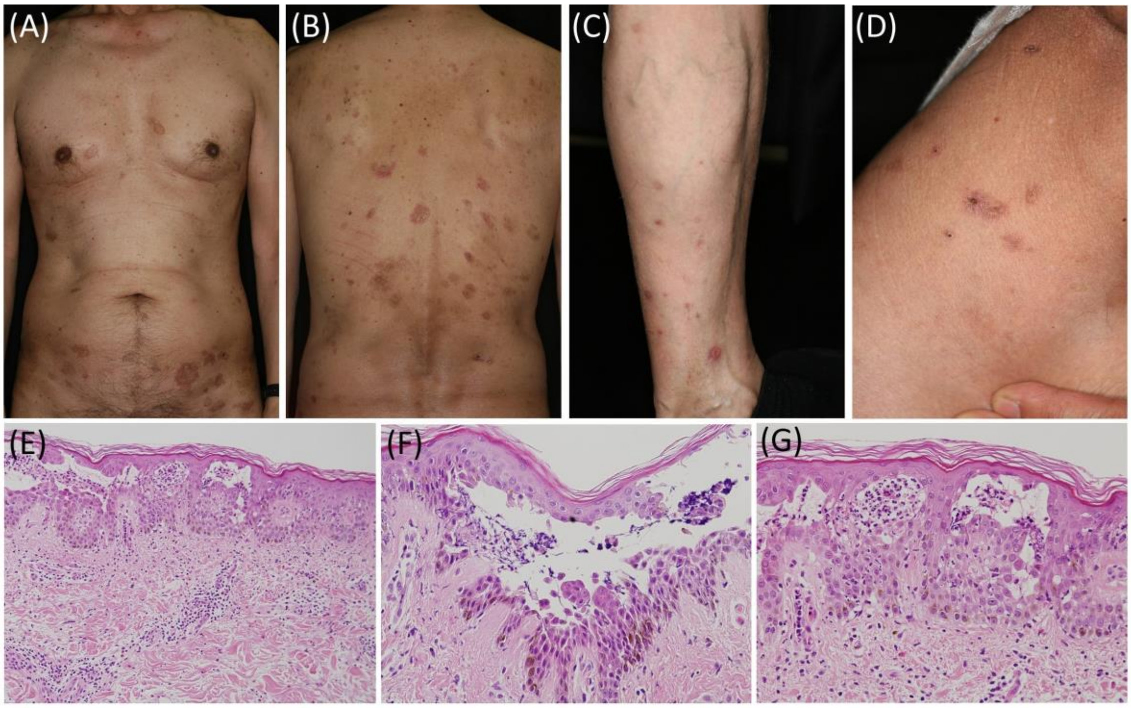

Clinical, histopathological and IF findings in case 23.

(A-D) Clinical features.   (E-G) Histopathological features.

Case 25 (FIGURE S4)

An 84-year-old Japanese male with past histories of pneumoconiosis and diabetes mellitus, had recurrent skin lesions on the axillae about 3 years ago, which lasted for about 2 years. Then, the patients developed annular erythematous skin lesions on the inguinal areas about 6 months ago, which were not responded to topical corticosteroids and oral antihistamines. The patient visited Ueo Dermatological Clinic, Saiki, Oita, Japan, in February 2013.

Physical examination revealed exudative annular erythemas with desquamation, as well as and vesicles and pustules on the peripheries of the erythemas, on the inguinal regions (Figure S4A,B) and minimum scaly erythemas on the axillae (Figure S4C). Laboratory tests showed normal results, except for slight elevations in serum IgG (1923 mg/dl, normal 820-1740) and HbA1C (7.0%, normal 4.3-5.8). Bacterial culture from lesional skin grew staphylococcus aureus.

Histopathology for a biopsy taken from lesional skin on the inguinal area revealed subcorneal blister filled with neutrophils and eosinophils in the epidermis and inflammatory infiltration of lymphocytes and neutrophils in the dermis (Figure S4D,E). Direct IF of skin biopsy from axilla revealed deposition of IgG and IgA to keratinocyte cell surfaces.

Regarding immunological tests, no positive reactivity was observed in IF of normal human skin and 1M NaCl-split-normal human skin, as well as IB of normal human epidermal extract, for both IgG and IgA antibodies.

ELISAs were negative for Dsg1 (index 13.34) but positive for Dsg3 (index 16.53) for IgG antibodies, and were negative for both Dsg1 (OD 0.026) and Dsg3 (OD 0.051) for IgA antibodies. IgG ELISAs were negative for Dsc1 (OD 0.124) and Dsc2 (OD 0.019) but positive for Dsc3 (OD 0.313). IgA ELISAs were negative for Dsc1 (OD 0.012) and Dsc2 (OD 0.004) but positive for Dsc3 (OD 0.159).

From these results, the patient was diagnosed as IGAD. The patient was treated with topical corticosteroids and PUVA therapy, but the skin lesions on the inguinal regions and axillae continued to recur for 5 year follow-up period (Figure S4F,G, clinical pictures taken in July 2016). Then, low dose of prednisolone with combination of tetracycline and niacinamide could suppress the skin lesions. The patient is currently controlled well only with nicotinamide.

FIGURE S4 (Case 25)

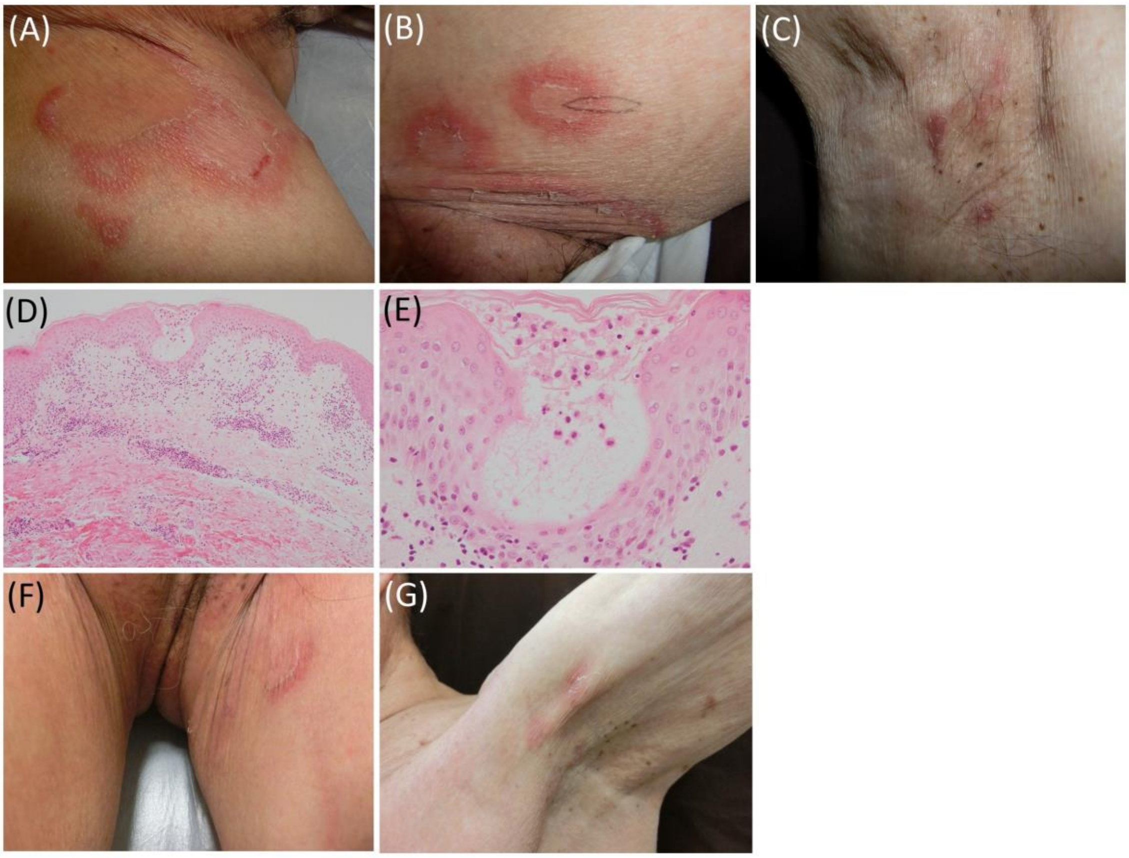

Clinical, histopathological and IF findings in case 25.

(A-C) Clinical features at the first visit. (D,E) Histopathological features.

(F,G) Current clinical features.

Case 27 (FIGURE S5)

A 39-year-old female had rheumathoid arthritis for 13 years under treatment of methotrexate 15 mg/week and folic acid 5 mg/week, which was added with PSL 10 mg/day and calcium carbonate 500 mg/day for the last 6 months. The patient developed blistering skin lesions with severe pruritus 3 month ago, which were poorly responsive to combination treatment of antibiotics and topical corticosteroids. The skin lesions were initially confined to the lower abdomen but gradually spread to other sites. The patient visited University Hospital Centre Zagreb, University of Zagreb School of Medicine, Zagreb, Croatia, in May 2013.

Physical examination revealed numerous annular erythemas and partially eczematoid skin lesions with vesicles, pustules, erosion and crusts on the periphery of the erythemas on the trunk and upper extremities, which suggested autoimmune bullous disease, particularly pemphigus herpetiformis (Figure S5A-D). Mucous membranes were spared. Staphylococcus aureus was isolated by bacterial culture of skin lesions, but fungus was negative. A complete blood counts showed iron deficiency anemia and eosinophilia (eosinophils 27%). Other laboratory studies and urine analysis showed normal values. Because of iron deficiency anemia, the patient underwent a gynecological examination that detected metrorrhagia for which IUD use was suggested.

Histopathology for skin biopsy revealed pustules containing mainly eosinophils without apparent acantholytic cells in the epidermis (Figure S5E). Direct IF detected IgG and IgA deposits to keratinocyte cell surfaces in the entire epidermis and deposits of C3 at basement membrane zone (BMZ).

Indirect IF of normal human skin detected IgG and IgA anti-cell surface antibodies at titers 1:80 (Figure S5F,G). Indirect IF of monkey esophagus also showed IgG and IgA anti-cell surface antibodies at titers 1:40 (Figure S5H,I). Indirect IF on 1M NaCl-split normal human skin showed negative results which excluded coexistance of subepidermal autoimmune bullous skin diseases.

IB of normal human epidermal extract did not detect any specific proteins, including the160 kDa Dsg1, 130kDa Dsg3 and the 110 kDa a-form and the 100 kDa b-form of Dsc for either IgG or IgA antiboides in the patient serum (Figure S5J).

IgG ELISAs were positive for Dsg1 (index 199.16) but negative for Dsg3 (index 5.91), and IgA ELISAs were positive for Dsg1 (OD 1.195) but negative for Dsg3 (OD 0.027). IgG ELISAs were positive for Dsc1 (OD 0.327) but negative for Dsc2 (OD 0.022) and Dsc3 (OD 0.028), while IgA ELISAs were negative for all Dsc1 (OD 0.019), Dsc2 (OD 0.004) and Dsc3 (OD 0.005).

Based on the results of direct IF, indirect IF and ELISA studies, diagnosis of IGAD was made. Most of previously reported cases of IGAD showed good response to DDS and other sulfones, which could not used in our patient due to iron defficiency anemia. Instead, systemic corticosteroids in dose of 0.5mg/kg (equivalent to PSL) were administered, followed by maintenance dose of 10 mg/day with topical cortocosteroids. Because staphylococcus aureus was isolated from skin lesions, oral cephalexin was also administered (1g/day for 7 days). The treatments resulted in complete resolution of both skin lesions and pruritus. The patients occasionally showed mild recurrences triggered by ultraviolet radiation.

FIGURE S5 (Case 27)

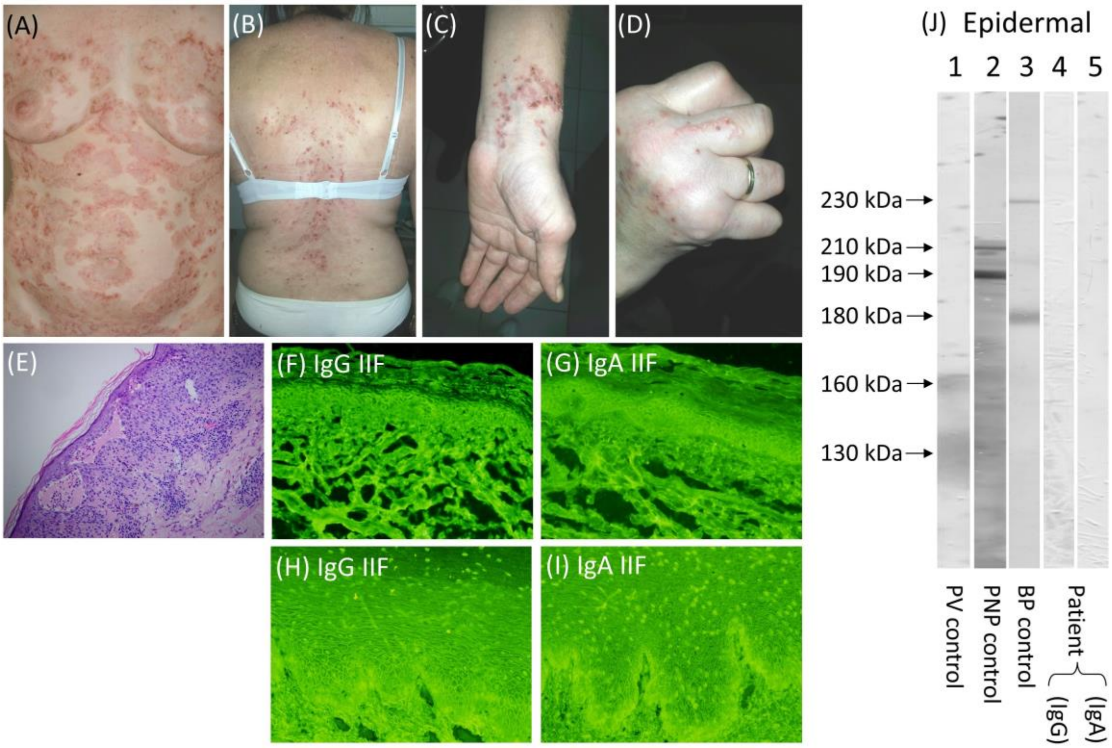

Clinical, IF and immunoblotting findings in case 27.

(A-D) Clinical features. (E) Histopathological feature. (F,G) The results of indirect IF of normal human skin for IgG (F) and IgA (G) antibodies. (H,I) The results of indirect IF of monkey esophagus for IgG (H) and IgA (I) antibodies. (J) The results of immunoblotting of normal human epidermal extract. IgG antibodies in control pemphigus vulgaris (PV) serum reacted with the 160 kDa Dsg1 and the 130 kDa Dsg3 (lane 1), IgG antibodies in control paraneoplastic pemphigus (PNP) serum reacted with the 210 kDa envoplakin and the 190 kDa periplakin (lane 2) and IgG antibodies in control bullous pemphigoid (BP) serum reacted with the 230 kDa BP230 and the 180 kDa BP180 (lane 3). The serum of case 27 did not show any positive reactivity for both IgG (lane 4) and IgA (lane 5) antibodies.

Case 29 (FIGURE S6)

A 12-year and 9 month-old Caucasian boy developed skin lesions 7 weeks ago, which gradually increased and spread. The patient visited Children's Hospital Colorado, Denver, Colorado, US, in January 2015.

Physical examination revealed erythematous skin lesions with vesicles and pustules on the scalp, face, posterior neck, chest and pubis (Figure S6A-C). There were no mucosal involvements. Severe pain and pruritus disturbed sleep of the patient. The patient had neither fever nor arthralgia. Swabs of skin lesions showed Staphylococcus aureus on bacterial culture, but did not show positive results for herpes simplex and herpes zoster.

Histopathology of lesional skin showed extensive pustules of prominent neutrophils and a few eosinophils with acantholytic cells in the upper epidermis (Figure S6D,E). Similar pustules were also seen in the mid-epidermis in less extent (Figure S6D). Suprabasal acantholysis was seen both in the epidermis and in the follicular epithelia (Figure S6D). Perivascular mixed cell infiltration was seen in the dermis (Figure S6D).

Direct IF showed deposition of both IgG and IgA to cell surfaces in the lower epidermis, while C3 was deposited to both cell surfaces in the lower epidermis and epidermal BMZ (Figure S7F,G).

Indirect IF of normal human skin sections detected IgA, but not IgG, anti-cell surface antibodies. Indirect IF of monkey esophagus showed positive IgA, but not IgG, reactivity to cell surfaces in the lower epithelia. IB of normal human epidermal extract revealed IgA reactivity with the 110 kDa a-form and the 100 kDa b-form of Dsc, but showed no positive IgG antibodies.

IgG ELISAs were negative for Dsg1 (index 0.7) but positive for Dsg3 (index 14.3), and IgA ELISAs were negative for Dsg1 (OD 0.02) but positive for Dsg3 (OD 1.044). IgG ELISAs were negative for Dsc1 (OD 0.04) but positive for Dsc2 (OD 0.098) and Dsc3 (OD 0.431), while IgA ELISAs were negative for Dsc1 (OD 0.011) but positive for Dsc2 (OD 0.095) and Dsc3 (OD 0.974).

The diagnosis of IGAD was made. Treatment of oral cephalexin, amoxicillin and terbinafine with topical antibiotics and topical antifungals was not effective. Subsequent oral PSL 40mg/day did not improve the skin lesions, either. Addition of dapsone 75mg/day to PSL 40mg/day was effective and quickly suppressed most skin lesions (Figure S7H-J). All skin lesions cleared and PSL quickly tapered off.

However, about one year later, skin lesions severely flared on the similar sites of the body. Histopathology and direct IF for the second biopsy also showed the same results

as those in the first biopsy. Restart of PSL 40mg/day and dapsone 100-150 mg/day was partially effective. Addition of mycophenolate mofetile was slightly effective. Then, rituximab 1gm twice nearly completely resolved the skin lesions.

However, one month later, dapsone and mycophenolate mofetile stopped, because of severe neutropenia. One month later, skin lesions flared. Then, dapsone 100 mg restarted and was partially effective. Isotretinoin was only partially effective. Six months later, the patient underwent second course of rituximab 1g twice, which did not completely cleared the skin lesions.

In January 2015, the patients still showed erosive lesions on the oral mucosae and the lips and erythematous skin lesions on the pubis and groins (Figure S6K,L). Then, the third course of rituximab 1g twice was performed. At this time, CT scan for the chest, abdomen and pelvis showed no abnormal results. In May 2016, the patient is 17 years and 6 month old and had erythematous plaques on the groins without mucosal lesion under PSL 40 mg and 30 mg alternating day, dapsone 150 mg/day, doxycycline 200 mg/day, mycophenolate mofetile 3000 mg/day, with topical corticosteroids.

FIGURE S6 (Case 29)

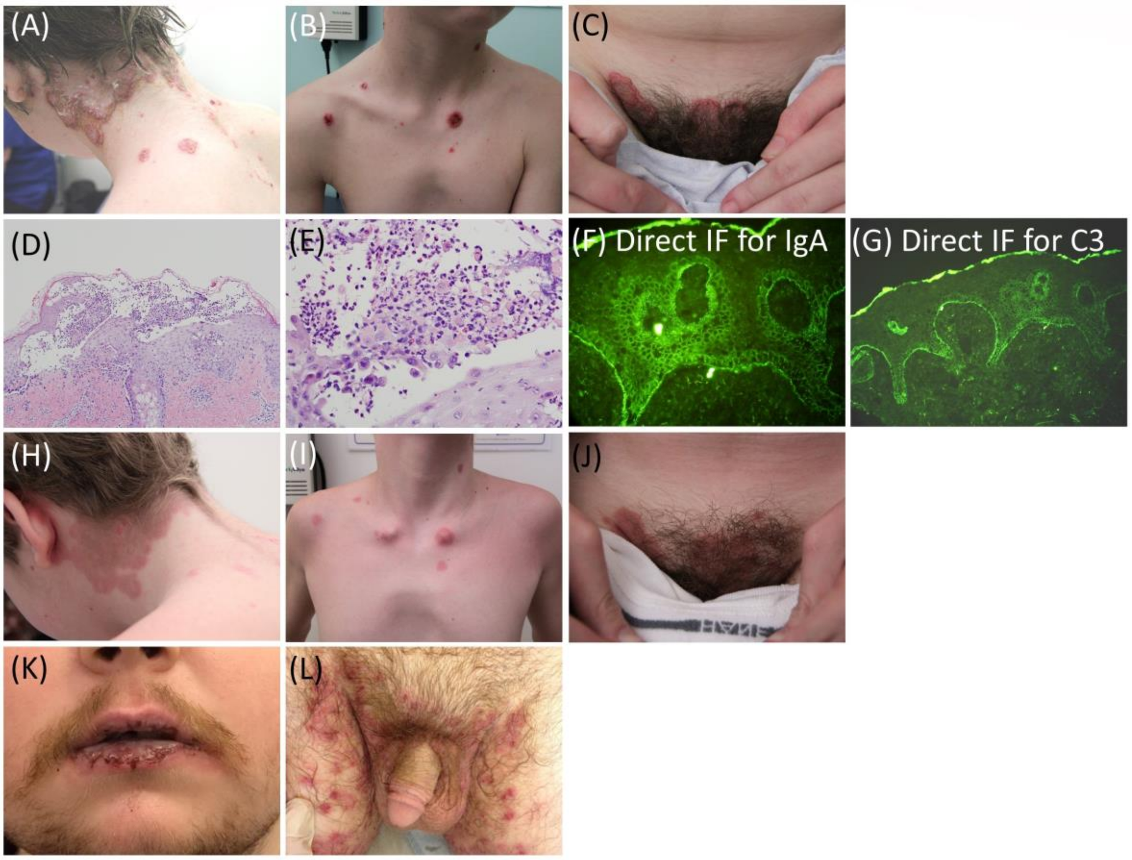

Clinical, histopathological and IF findings in case 29.

(A-C) Clinical features at the first visit. (D,E) Histopathological features. (F,G) The results of direct IF for IgA (F) and C3 (G). (H-J) Clinical features for improved skin lesions after the original DDS therapy. (K,L) Clinical features on January 2015.

FIGURE S7 The schematic presentation of class switch recombination (CSR) for antibody class switching from IgM/IgD to IgG and IgA classes

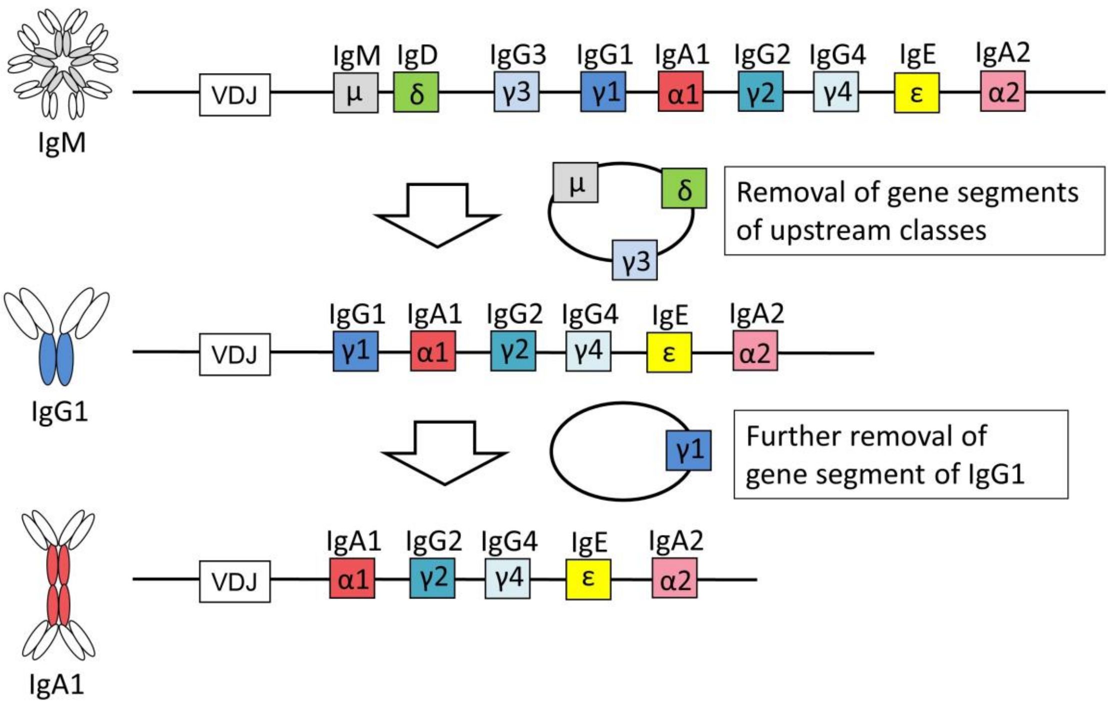

CSR occurs through a genomic rearrangement within constant region locus of immunoglobulin heavy chain, where gene segments of all immunoglobulin classes are tandemly located downstream of the VDJ variable region locus (upper panel). The upstream classes are looped out through the CSR and the downstream class is docked into the VDJ region (middle panel). Although a class has been considered to be switched from IgM/IgD producing B-cells to IgG, IgA and IgE producing B-cells, a recent study suggested more variable class switch pathways, including pathway from IgG1 to IgA1 (lower panel).

TABLE S1 Whole clinical and histopathological data for the 30 IGAD patients

Cases	Ages (years)	Genders	Countries	Clinical diagnoses	Final diagnoses	Past histories	Disease durations	Previous oral drugs	Sites of lesions	Clinical features	Histopathological findings	Treatments	Responses (prognoses)
1	20	F	Japan	IGAD/PNP	IGAD				Oral(+), limbs, nails	Annular erythema, blister, vesicle, erosion		PSL	Not very effective
2	11	M	USA	IAD	IGAD+LAGBD				Oral(+), lips, limbs				
3	39	F	Poland	IGAD	IGAD	Uterine cancer	１ year			Annular erythema, erosion			
4		M	Japan	PV	IGAD		1 month		Oral(+), lips, limbs, palms, soles	Erythema, blister, erosion	Pustule, middle epidermis, neutrophil		
5	37	M	Japan	IGAD	IGAD		2 years	minocycline	Oral(+), esophagus, trunk	Erosions　Annular erythema, blister, erosion, pustule	Pustule, intraepidermal, neutrophil	PSL+minocycline	Relatively effective
6	81	F	Japan	IGAD	IGAD	Cholangiocarcinoma, diabetes mellitus	2 months	Glimepiride	Trunk, limbs	Annular erythema, blister, flaccid blister, itch(+)	Blister, subcorneal, neutrophil		
7	71	M	Spain	IGAD	IGAD	Myasthenia gravis	2 years	antihistamins	Whole body, oral (lichen planus)	Erythema, blister, pustule, itch(+)	Blister, intraepidermal		
8	43	F	Japan	IGAD	IGAD		1 year		Head, whole body	Annular erythema, blister, vesicle	Blister, subcorneal, neutrophil	PSL<br>DDS	Effective<br>Not effective
9	49	F	Japan	PV	IGAD	Pemphigus vulgaris	3 years	PSL		Annular erythema, blister			
10	25	F	Spain	IGAD	IGAD		4 months		Oral(-), trunk	Annular erythema, blister, erosion	Pustule, intraepidermal, acantholysis(+)	PSL30g, IVIG, DFPP, steroid pulse	Not effective (died)
11	79	F	Japan	MMP/PNP	IGAD+LAGBD		4 months		Nasal mucosa, lips, eyelids, trunk	Erosion, crust	Blister, all epidermis, neutrophil		
12	64	M	Australia	IGAD	IGAD	Lymphoma	18 months		Whole body	Erythroderma, blister, pustule	Pustule, lower epidermis, neutrophil, acantholysis(+)		
13	75	F	Japan	PF/PH	IGAD				Olal(-), trunk, limbs	Tense blister, vesicle, pustule, pigmentation, itch(+)	Blister, intraepidermal		
14	61	F	Japan	IAD	IGAD		3 years		Face, trunk	Pustule, erythema, blister, crust	Pustule, middle epidermis, neutrophil		
15		M	Japan	IAD	IGAD	Sweet disease	1 month		Oral(+), trunk, limbs		Pustule, intraepidermal, neutrophil		
16			Hungary	IAD/LAD	IGAD	Harada disease							
17	58	F	Poland	IGAD	IGAD	Breast cancer, hypertension			Oral(+), limbs, shin, hand、fingers			PSL 30g + DDS 100mg	Effective
18	81	M	Japan	IAD/dermatitis herpetiformis	IGAD+linear IgA bullous dermatosis	Ni Allergy, IgA nephropathy, aquired ichthyosis			Trunk, limbs	Erythema, blister, tense blister	Blister, spongiosis, upper epidermis, neutrophil, lymphocyte		
19	55	M	India		IGAD		5 years		Face, trunk, limbs, palms, soles	Urticarial erythema, blister, flaccid blister, hypopion, pitmentation, itchi(+)	Blister, upper epidermis, acantholysis(-)	PSL+cyclophosphamidDDS	Effective<br>Not effective
20	56	M	Japan	IAD	IGAD		1 year	Chlorpheniramine, betamethasone		Annular erythema	Spongiosis	PSL+DDS	Effective
21	55	M	India	IGAD	IGAD								
22	80	F	Japan	IAD	IGAD	Sjogren syndrome	10 days		Trunk, limbs	Annular erythema, blister, vesicle	Pustule, eosinophilic spongiosis, all epidermis, eosionphi, eosinophil	PSL 20mg	Effective
23	64	M	Japan	IGAD	IGAD	None	10 years	Epinastine	Trunk, limbs	Erythema, blister, itch(+)	Blister, intraepidermal, eosinophil		
24	66	F	Japan	IGAD	IGAD		1 month		Oral(+), ocular hyperemia, head, trunk, limbs	Blister, flaccid blister, erosion	Bliseter, intraepidermal, neutrophil, eosinophil		
25	84	M	Japan	IAD	IGAD				Groins, axillae	Annular erythema, blister, pustule	Pustule, upper epidermis, neutrophil, pneumoconiosis, diabetes mellitus		
26	50	M	Japan	Pemphigus vegetans	IGAD+bullous pemphigoid		2 months		Oral(+), limbs, feet	Blister, erosion, pustule, vegetation, itch(+)	Pustule, entrapidermal, eosinophil	PSL<br>Minocycline	Not effective<br>Effective
27	39	F	Croatia	Pemphigus herpetiformis	IGAD	Reumatoid arthritis	5 months	Methotrexate, PSL	Trunk, limbs、Upper limbs	Erythema, vesicle, erosion, crust, itch(+)	Pustule, intraepidermal, eosinophil	PSL 0.5mg/kg/day + cephalexin 1g	Effectvie<br>(slight reccurrence)
28	65	M	USA	IGAD	IGAD		2 weeks	PSL 1mg/kg /day	Trunk, limbs	Erythema, vesicle, erosion, crust	Blister, upper epidermis, acantholysis(+)	DDS 75mg<br>PSL 60mg	Effective<br>Effective
29	12	M	USA	IAD	IGAD		7 weeks		Whole body	Blister, erosion, pustule	Pustule, upper epidermis, neutrophils, acantholysis(+), blister lower epidermis	PSL<br>DDS<br>Mycophnolate mofetil<br>Rituximab<br>Isotretinoin	Not effective<br>Slightly effective<br>Not effective<br>Effective<br>Not effective
30		F	Japan	IGAD	IGAD				Oral(+), ocular mucosa, vulvar mucosa, head	Erythema, erosion, pustule, ulcer, itch(+)	Pustule, intraepidermal, eosinophil	PSL 10mg<br>DDS 7T	Not effective<br>Not effective

TABLE S2 Whole immunological data for the 30 IGAD patients

Cases	Direct immunofluorescence						Noraml human skin IIF				Monkey esophagus IIF				IM NaCl--split IIF				IB		ELISAs										COS-7		BMZ data
	IgG		IgA		C3		IgG		IgA		IgG		IgA		IgG		IgA		epidermal		IgG		IgA		IgG			IgA			IgG	IgA	
	BMZ	CS	BMZ	CS	BMZ	CS	BMZ	CS	BMZ	CS	BMZ	CS	BMZ	CS	epi-dermal	dermal	epi-dermal	dermal	(G)	(A)	Dsg1 cut off 14	Dsg3 cut off 7	Dsg1 cut off 0.15	Dsg3 cut off 0.15	Dsc1 cut off 0.2	Dsc2 cut off 0.07	Dsc3 cut off 0.12	Dsc1 cut off 0.123	Dsc2 cut off 0.048	Dsc3 cut off 0.074	Dsc1 Dsc2 Dsc3	Dsc1 Dsc2 Dsc3	
1	-	-	-	+ all			-	+ x40	-	+ x40									100(+) 110(+) 210(+) 250(+)	100+ 110+	+	+	+	+	Not done						-	-	
2	+	-	+	-			+ >x160	+ >x160	-	+ >x160					+ >x40	-	+ >x40	-	-	-	15.99	53.15	0.111	1.642	0.288	0.167	0.287	0.637	0.234	0.72	-	-	LAD-1 (IgG) +
3							-	+ x40	-	+ >x160									-	-	242.98	6.24	2.702	0.097	0.403	0.058	1.120	0.173	0.029	0.023	-	-	
4	-	+	-	+	-	+	-	+ >x160	-	+ x40					-	-	-	-	-	-	22.2	6.61	0.395	0.082	0.035	0.048	0.021	0.037	0.05	0.016	-	-	
5	-	+	-	+			-	+ x40	-	+ x40									130+	-	0.87	142.52	0.03	0.238	0.072	0.042	0.056	0.036	0.019	0.025	-	-	
6	-	+	-	+	-	+	-	+>x160	-	+ >x160									-	-	155.05	0.31	0.479	0.037	0.175	0.031	0.03	0.055	0.013	0.019	-	-	
7							-	-	-	-	-	+	-	+					-	-	82.27	-7.47	1.739	0.004	0.01	0.004	0.008	0.194	0.02	0.01	-	-	
8	-	+ up	-	+ up							-	+	-	+					-	-	207.2	1.2	140.5	1.4	0.077	0.016	0.035	0.047	0.010	0.009	-	-	
9							-	-	-	+ x10	+	+	+	+	-	-	-	-	-	-	178.74	41.42	0.15	1.728	0.206	0.374	0.135	0.282	0.231	0.187	-	Dsc1 +	
10	-	+ all	-	+ all			-	-	-	+ x10	-	+	-	+					-	-	104.84	13.11	0.198	-0.023	0.02	0.014	0.008	0.055	0.022	0.016	-	-	
11	Tissue damaged						+ x40	-	+ x40	+ x40					-	-	+ >x40	-	230+	-	207.3	175.6	0.687	0.577	0.598	0.162	0.152	0.06	0.051	0.021		Dsc3+	Epi-IB(IG) :BP230 + BP180NC16a-IB (IgG) +
12	-	+ low	-	+ low		+ low	-	-	-	-	-	-	-	-	-	-	-	-	-	-	0.49	0.00	0.07	-0.167	0.043	0.023	2.271	0.106	0.028	2.472	Dsc3 ++	Dsc 3+	
13								+ x40		+ x10	-	+	-	+	-	-	-	-	160+/-	-	+	-	1.892	0.1	-	+	+	0.841	0.016	0.015	-	-	
14	-	-	-	-			-	-	-	-	-	+	-	+					-	-	-	-	-0.124	0.285	0.06	0.212	0.027	0.044	0.013	0.008	-	-	
15							-	-	-	-	-	+	-	+	-	-	-	-	-	-	29.96	21.48	0.321	0.217	0.05	0.024	0.029	0.094	0.056	0.089	-	-	
16							-	-		+ x40	-	+	-	+	-	-	-	-	-	-	42.5	0.24	2.238	0.026	0.116	0.011	0.178	0.053	0.001	0.003	-	-	
17		++		++			-	+ x10	-	-	-	+	-	-					130+	-	61.03	145.77	0.968	0.88	0.031	0.021	0.02	0.046	0.053	0.054	-	-	
18	(-)	(-)	(-)	(-)			-	+	-	+	-	+	-	+	-	-	+>x40	-	-	-	0.63	0.48	0.147	0.033	0.026	0.019	0.083	0.009	0.002	0.016	-	-	All negative
19	-	++	-	+				+ >x160	-	-	+	+	+	+	-	-	-	-	160+	-	218.99	0.2	0.385	0.028	0.109	0.022	0.024	0.016	0.001	-0.004	-	-	
20	-	-	(-)	(+) all			-	-	-	-	-	+	-	+					-	-	1.19	0.34	0.033	0.009	0.015	0.008	0.007	0.061	0.048	-0.005	-	-	
21	-	+	-	+			-	+ >x160	-	+					-	-	-	-	160+	-	+	-	+	-	0.12	0.03	0.043	-0.002	-0.008	-0.009	-	-	
22	-	+	-	-	-	+	-	-	-	-	-	-	-	-					-	-	-	-	-0.029	-0.073	0.454	0.016	2.254	0.057	0.016	0.312	-	-	
23	-	+	-	+			-	+ x10	-	+ x40	-	+	-	+					-	-	8.99	8.07	0.193	0.011	0.097	0.007	0.015	0.064	0.033	0.011			
24	-	+ low	-	+ low				+ >x160		+ x10					-	-	-	-	-	-	63.36	149.86	0.264	0.418	0.089	0.145	0.06	0.015	0.008	0.001	-	-	
25	-	+	-	+			-	-	-	-					-	-	-	-	-	-	13.34	16.53	0.026	0.051	0.124	0.019	0.313	0.012	0.004	0.159			
26							-	+ x10	-	+ x10					+ >x40	-	-	-	130+	130+	1.61	26.65	0.012	0.45	0.068	0.014	0.041	0.033	0.014	0.027	-	-	BP180-NC16a-IB I(G) + BP180 ELISA (IgG) 19.98 +
27	-	+ all	-	+ all	+	-	-	+ x40	-	+ x40	-	+	-	+	-	-	-	-	-	-	199.16	5.91	1.195	0.027	0.327	0.022	0.028	0.019	0.004	0.005			
28	-	+	-	+			-	+ x40	-	+ x40	-	-	-	-	-	-	-	-	-	-	126.42	1.22	0.227	0.014	0.032	0.01	0.974	0.053	0.009	0.005			
29	-	+ low	-	+ low	+	+ low	-	-	-	+ x40	-	-	-	+ lower					-	100+ 110+	0.7	14.3	0.02	1.044	0.04	0.098	0.431	0.011	0.095	0.974			
30	-	+	-	+			-	+	-	+ x40									-	-	2.01	9.92	0.061	2	0.089	0.016	0.018	0.098	0.019	0.026			

IIF: indirect immunofluorescence. IB: immunoblotting. CS: cell surface. BMZ: basement membrane zone. COS-7: COS-7 cell cDNA tranfection method.

TABLE S3 Clinical and final diagnoses

(A) Clinical diagnoses	No.
IGAD	12
IAD	9
IAD/dermatitis herpetiformis	1
IAD/linear IgA dermatosis	1
Pemphigus vulgaris	2
Pemphigus vegetans	1
Pemphigus foliaceus/PH	1
PNP/MMP	1
none	2
Total	30

(B) Final diagnoses	No.
IGAD	26
IGAD+bullous pemphigoid	1
IGAD + linear IgA dermatosis	1
IGAD+LAGBD	2
Total	30

IGAD: Intercellular IgG/IgA dermatosis.
IAD: intercellular IgA dermatosis.
PH: pemphigus herpetiformis
PNP: paraneoplastic pemphigus
MMP: mucous membrane pemphigoid
LAGBD: linear IgA/IgG bullous dermatosis

TABLE S4 Treatments and the responsiveness to them

Treatments: no. of cases		No. of cases responsive to the therapies
PSL	7	3
DDS	5	2
Minocycline	1	1
PSL+DDS	1	1
PSL+minocycline	1	1
PSL+cyclophosphamide	1	1
IVIG	1	0
DFPP	1	0
Steroid pulse therapy	1	0

PSL: oral prednisolone.
IVIG: intravenous immunoglobulins
DFPP: double filtration plasmapheresis

TABLE S5 Results of immunoblotting of normal human epidermal extract

IgG	Positive	Total	Positive rates
250 kDa desmoplakin I	1	30	3.3%
230 kDa BP230	1	30	3.3%
210 kDa envoplakin	1	30	3.3%
190 kDa periplakin	0	30	0%
160 kDa Dsg1	3	30	10.0%
130 kDa Dsg3	3	30	10.0%
110 kDa a-form of Dsc	1	30	3.3%
100 kDa b-form of Dsc	1	30	3.3%

IgA	Positive	Total	Positive rates
250 kDa desmoplakin I	0	30	0%
230 kDa BP230	0	30	0%
210 kDa envoplakin	0	30	0%
190 kDa periplakin	0	30	0%
160 kDa Dsg1	0	30	0%
130 kDa Dsg3	1	30	3.3%
110 kDa a-form of Dsc	2	30	6.7%
100 kDa b-form of Dsc	2	30	6.7%

SUPPLEMENTARY ACKNOWLEDGMENTS

We gratefully appreciate following dermatologists for their kind requests for our diagnostic studies and for generously sending us the information and the sera of their important cases with IGAD.

Dr. Fenella Wojnarowska, Department of Dermatology, Oxford Radcliffe Hospital, Oxford, UK.
Dr. Cezary Kowalewski, Department of Dermatology and Immunodermatology, Medical University of Warsaw, Warsaw, Poland.
Dr. Atsuko Adachi, Department of Dermatology, Kakogawa Prefectural Hospital, Kakogawa, Hyogo, Japan.
Dr. Tetsuko Nakamura, Department of Dermatology, Fukagawa City Hospital, Fukagawa, Hokkaido, Japan.
Dr. Yasuhiro Kawachi, Department of Dermatology, Tsukuba University School of Medicine, Tsukuba, Ibaraki, Japan.
Dr. Javier Pedraz, Department of Dermatology, La Princesa University, Madrid, Spain.
Dr. Hiroyoshi Hoshino, Department of Dermatology, Hiratsuka City Hospital, Hiratsuka, Kanagawa, Japan.
Dr. Nobuyasu Mayuzumi, Department of Dermatology, Juntendo University School of Medicine, Bunkyo-ku, Tokyo, Japan.
Dr. Afreca Jaurez, Department of Dermatology, La Princesa University, Madrid, Spain.
Dr. Kanade Kawasaki (Temporin), Department of Dermatology, St. Marianna University School of Medicine, Kawasaki, Kanagawa, Japan.
Dr. Suzan Foley, Department of Dermatology, Princess Alexandra Hospital, Australia.
Dr. Eri Yoshimura, Department of Dermatology, Kyushu Medical Center, Chuo-ku, Fukuoka, Japan.
Dr. Emiko Okada, Department of Dermatology, Keio University School of Medicine, Shinjuku, Tokyo, Japan.
Dr. Klaudia Preisz and Dr. Antal Blazsek, Budapest, Hungary.
Dr. Miho Ueda, Department of Dermatology, Kyoto University School of Medicine, Sakyo-ku, Kyoto, Japan.
Dr. Amrinder J, Kanwar, Department of Dermatology, Venereology and Leprology, Postgraduate Institute of Medical Education and Research, Chandigarh, India.
Dr. Hirai Aiko, Department of Dermatology, National Defense Medical College, Tokorozawa, Saitama, Japan.
Dr. Tomoko Inoue, Department of Dermatology, Japan Community Health Care Organization Osaka Hospital, Fukushima-ku, Osaka, Japan.
Dr. Yuri I. Bunimovich, Department of Dermatology, University of Pittsburgh Medical Center, Pittsburgh, Pennsylvania, US.
Dr. Kyoko Yoneyama, Department of Dermatology, Chiba University School of Medicine, Chuo-ku, Chiba, Japan.
